# Effect of surface treatments on biaxial flexural strength, fatigue resistance, and fracture toughness of high versus low translucency zirconia

**DOI:** 10.1186/s12903-022-02431-8

**Published:** 2022-09-19

**Authors:** Alaaeldin Elraggal, Moustafa Aboushelib, Islam M. Abdel Raheem, Rania R. Afifi

**Affiliations:** 1 Conservative Dentistry Department, Faculty of Dentistry, Alexandria University, Alexandria, Egypt; 2 Dental Biomaterials Department, Faculty of Dentistry, Alexandria University, Alexandria, Egypt

**Keywords:** Zirconia, Translucency, Cubic, Tetragonal, Fatigue, Fracture toughness, SEM, Grain size

## Abstract

**Background:**

Mechanical surface treatments can deteriorate the mechanical properties of zirconia. This study evaluated and compared the biaxial flexural strength, fracture toughness, and fatigue resistance of high translucency (HT) to low translucency (LT) zirconia after various mechanical surface treatments.

**Methods:**

Four hundred eighty zirconia discs were prepared by milling and sintering two HT (Katana and BruxZir) and LT (Cercon and Lava) zirconia blocks at targeted dimensions of 12 mm diameter × 1.2 mm thickness. Sintered zirconia discs received one of the following surface treatments: low-pressure airborne particle abrasion (APA) using 50 µm alumina particles, grinding using 400 grit silicon carbide paper, while as-sintered specimens served as control. Internal structure and surface roughness were evaluated by scanning electron microscope (SEM) and a non-contact laser profilometer, respectively. Half of the discs were tested for initial biaxial flexural strength, while the rest was subjected to 10^6^ cyclic fatigue loadings, followed by measuring the residual biaxial flexural strength. Fractured surfaces were examined for critical size defects (c) using SEM to calculate the fracture toughness (K_IC_). The effect of surface treatments, zirconia type, and cyclic fatigue on the biaxial flexural strength was statistically analyzed using three-way analysis of variance (ANOVA) and Tukey HSD post hoc tests (α = 0.05). Weibull analysis was done to evaluate the reliability of the flexural strength for different materials.

**Results:**

The initial biaxial flexural strength of LT zirconia was significantly higher (*p* < 0.001) than that of HT zirconia in all groups. While low APA significantly increased the biaxial flexural strength of LT zirconia, no significant change was observed for HT zirconia except for Katana. Surface grinding and cyclic fatigue significantly reduced the flexural strength of all groups. High translucency zirconia reported higher fracture toughness, yet with lower Weibull moduli, compared to LT zirconia.

**Conclusion:**

LT zirconia has higher biaxial flexural strength, yet with lower fracture toughness and fatigue resistance, compared to HT zirconia. Low-pressure APA has significantly increased the biaxial flexural strength in all zirconia groups except BruxZir. Grinding was deteriorating to biaxial flexural strength and fracture toughness in all zirconia types. Cyclic fatigue has significantly decreased the biaxial flexural strength and reliability of HT and LT zirconia.

## Background

Yttria-tetragonal zirconia polycrystal (Y-TZP) has been the most favorable bioceramic material used in dental practice due to its optimum mechanical properties [1]. Zirconia is a polymorphous material that exists in three crystalline structures at different temperatures [2]. When heated to 2680 °C, zirconia takes the cubic form. When the material is cooled down to 2370 °C, it has a tetragonal crystalline structure. Further cooling to 1170 °C and till room temperature, zirconia has its final stable crystalline form (monoclinic) [3]. Tetragonal to monoclinic phase (t–m) transformation is usually accompanied by a 4–5% volumetric expansion of zirconia due to distortion in the shape of tetragonal crystals leading to cracking of zirconia [4]. The addition of stabilizing oxides (MgO, CeO_2_, or Y_2_O) secures the tetragonal crystalline form of zirconia at room temperature and maintains its highest mechanical properties [5]. Zirconia was found to exhibit its maximum mechanical properties at its tetragonal structure represented in high flexural strength that exceeded 900 MPa, and fracture toughness of 5.5–7.4 MPa m^1/2^ [6]. However, when a crack is induced within zirconia, it creates enough pressure that makes the crystals surrounding the crack tip transform into a monoclinic phase. As a result, a slight volume increase of these crystals generates favorable compressive stresses around the crack’s direction and pins it down from propagating any further in a mechanism known as transformation toughening or phase transformation toughening [7].

Y-TZP has a submicroscopic grain size in the range of 0.2–0.5 µm and exhibits high opacity because of the inherent birefringence of non-cubic zirconia phases, resulting in light scattering at grain boundaries, pores, and additive inclusions [8, 9]. The opaque nature of Y-TZP has limited its use as a framework substructure to be essentially veneered with more esthetic glass–ceramics [10, 11]. Chipping and delamination of the veneering ceramic were some of the most clinically encountered problems of veneered Y-TZP frameworks [12]. It was reported that 15% of Y-TZP restoration replacement occurred due to delamination and 20% due to chipping after 5 years of clinical follow-up [13].

The introduction of monolithic or, as known, a high translucency (HT) zirconia restoration with improved optical properties has dispensed the need for the veneer layer [9]. Elimination of the veneering ceramic was a direct advantage that simplified fabrication technique, and it also dramatically reduced time and production cost [14]. Full anatomical zirconia restorations were proposed in the posterior region, especially with limited interocclusal space [15–17]. The HT zirconia has an exceptionally optimized translucency allowing light to penetrate through the material. This was achieved through increasing the grain size, optimizing grain boundary region, and reducing alumina content by incorporating higher Yttria content to produce partially stabilized zirconia, 4 mol% (4Y-PSZ) or 5 mol% (5Y-PSZ), with increased amounts of nonbirefringent cubic phase [8]. Diminishing the opacity was achieved by one or more of these mechanisms; sintering additives (typically alumina) [18], reduction of oxygen vacancies, pores, and defects as well as a controlled sintering environment (i.e. pressure and temperature) [19]. Low translucency zirconia generally has higher mechanical properties than HT zirconia; however, the enhanced optical properties of the latter made it more suitable to use in monolithic fixed restorations [20].

Different surface treatments such as APA and grinding are essential routine steps for better resin bonding to zirconia. However, these surface treatments can deteriorate the mechanical properties of Y-TZP and Y-PSZ and possibly induce surface flaws and microcracks that can propagate under occlusal loads leading to a catastrophic failure [12, 21]. Occlusal loads are far below the flexural strength of zirconia. However, with pre-existing surface defects, intermittent occlusal forces may lead to the propagation of those cracks and eventually lead to a fracture [22, 23]. Fracture toughness measures the material resistance to crack propagation; hence it could be affected by the magnitude of surface flaws or cracks that are induced by different mechanical surface treatments [24]. The effect of APA and grinding on the mechanical properties of HT zirconia has been less studied compared to LT zirconia [25]. Therefore this study aimed to evaluate the effect of low APA and surface grinding on biaxial flexural strength, fatigue resistance, and fracture toughness of HT versus LT zirconia frameworks. The null hypothesis tested was that different surface treatments will not affect the biaxial flexural strength and fracture toughness of either HT or LT zirconia.

## Materials and methods

### Preparation of zirconia specimens

Two LT 3 mol% Y-TZP zirconia (Cercon base, Dengudent, Hana Wolfgang, Germany, and Lava Frame, 3 M ESPE, Germany) and two HT 4 mol% Y-PSZ zirconia milling blocks (Katana HT, Kuraray Noritaki Dental Inc, Japan and BruxZir, Glidewell, USA) (Table 1) were milled, in their green state, into a total of 480 discs (14.5 mm × 1.5 mm) (n = 120/group) using a precision cutting machine (Isomet 5000, Buehler, Lake Bluff, Ill, USA). The discs were sintered according to the manufacturer’s recommendations at sintering temperatures of 1450 °C, 1500 °C, 1550 °C, and 1580 °C for Cercon Base, Lava Frame, Katana HT, and BruxZir, respectively, and a holding time for 2 h. The final dimensions of zirconia specimens were approximately 12 mm in diameter and 1.2 mm in thickness, due to ≈20% volumetric sintering shrinkage, following the recommendations of (ISO: 6872:2015) [22]. After sintering, one surface of each zirconia disc received one of different surface treatments (n = 40/subgroup); low-pressure airborne particle abrasion using 50 μm alumina particles at 0.5 bar using a sandblaster (AquaCare Twin, Velopex, Medivance Instruments Ltd., London, UK) or grinding using 400 grit silicon carbide paper (Waterproof Abrasive Paper, Daesung Abrasive Co., Seoul, Korea) using a metallographic polishing device under 300 g weight and water cooling, while the as-sintered specimens served as a control. The opposite surface of zirconia discs in all groups was left as polished.

**Table 1:** Zirconia types used in the study and their chemical composition

Material	Zirconia type	Chemical composition	Manufacturer
Cercon base	Low translucency	ZrO_2_ (92 wt%), Y_2_O_3_ 5.0 wt%, and HfO_2_: < 2.0 wt%	Degudent, Hana Wolfgang, Germany
Lava Frame	Low translucency	ZrO_2_ < 95.00%, Y_2_O_3_ < 5.00%, Al_2_O_3_ < 0.25%	3 M ESPE, Germany
Katana HT	High translucency	ZrO_2_ 90–95%, Y_2_O_3_ 5–8%, Other < 2%	Kuraray Noritaki Dental Inc, Japan
BruxZir	High translucency	ZrO_2_ 88%, Y_2_O_3_ 9%, Al_2_O_3_ 0.1% HfO_2_ 2.31%	Glidewell, USA

### Characterization of surface roughness and grain size

Surface roughness parameters (R_a_, R_p_, and R_v_) values were measured using a non-contact laser surface profilometer (Profilm 3D, Filmetrics Inc) where R_a_ is the mean surface roughness, R_p_ is the peak surface roughness, and R_v_ is the valley surface roughness. These parameters were expressed in µm. Grain size and grain boundary regions were evaluated using scanning electron microscopic examination (XL 30, Philips, Eindhoven, The Netherlands). The size of one hundred grains from each zirconia type was calculated on the obtained SEM images using computer software (Image analysis Java, NIH).

### Evaluation of initial biaxial flexural strength and fracture toughness

**Fig. 1:**
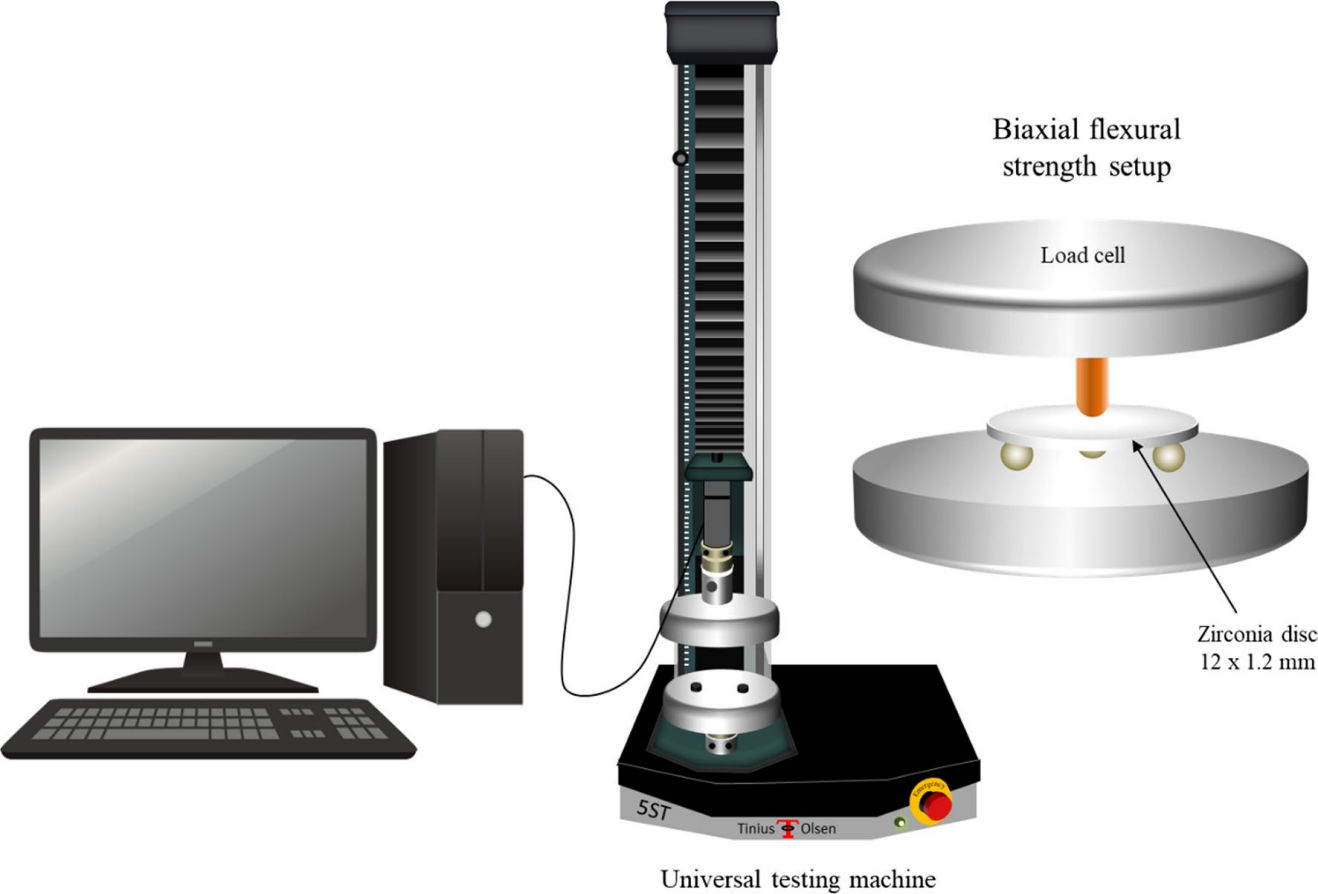
Schematic diagram showing the piston on three balls set-up for biaxial flexural strength testing. Zirconia discs were mounted so that the treated side was facing downwards. Specimens were then loaded to fracture using Tinius Olsen universal testing machine

Half of the zirconia discs (n = 20/subgroup) were loaded in a piston on three balls set-up for biaxial flexural strength testing (following ISO: 6872:2015) with the treated surface in tension to evaluate the initial biaxial flexural strength. Discs were loaded at a crosshead speed of 1 mm/min till failure using a universal testing machine (Tinius Olsen model no 5ST, Surrey, UK) (Fig. 1). The load cell (500 N) was calibrated using a digital scale (AcculabVicon VIC 711; Itin Scale Co., Brooklyn, NY), and the crosshead speed was observed using a digital traveling microscope (Millitron; Feinpruf Perthen GmbH, Gottingen, Germany). The biaxial flexural strength was then calculated using the following equations [26]:

1 $$Fs = \, - 0.{2837}\frac{{P \left( {X - Y} \right)}}{{d^{2} }}$$

where Fs is the biaxial flexural strength in MPa, P is the load to failure of specimen in (N), and d is the specimen thickness in mm. X and Y were calculated using the following equations:

2 $${\text{X}} = \, \left( {{1} + \, \nu } \right){\text{In}}\left( {{\raise0.7ex\hbox{${r2}$} \!\mathord{\left/ {\vphantom {{r2} {r3}}}\right.\kern-\nulldelimiterspace} \!\lower0.7ex\hbox{${r3}$}}} \right)^{2} + \left[ {\frac{1 - \nu }{2}} \right]\left( {{\raise0.7ex\hbox{${r2}$} \!\mathord{\left/ {\vphantom {{r2} {r3}}}\right.\kern-\nulldelimiterspace} \!\lower0.7ex\hbox{${r3}$}}} \right)^{2}$$

3 $${\text{Y}} = \, \left( {{1} + \, \nu } \right)\left[ {1 + In \left( {{\raise0.7ex\hbox{${r2}$} \!\mathord{\left/ {\vphantom {{r2} {r3}}}\right.\kern-\nulldelimiterspace} \!\lower0.7ex\hbox{${r3}$}}} \right)^{2} } \right] + (1 - \nu )\left( {{\raise0.7ex\hbox{${r1}$} \!\mathord{\left/ {\vphantom {{r1} {r3}}}\right.\kern-\nulldelimiterspace} \!\lower0.7ex\hbox{${r3}$}}} \right)^{2}$$

where ν is Poisson’s ratio (= 0.23); r1is the radius of the supported area of zirconia disc, r2 is the radius of the loaded area of the disc, r3 is the radius of the specimen, all of these are measured in mm.

**Fig. 2:**
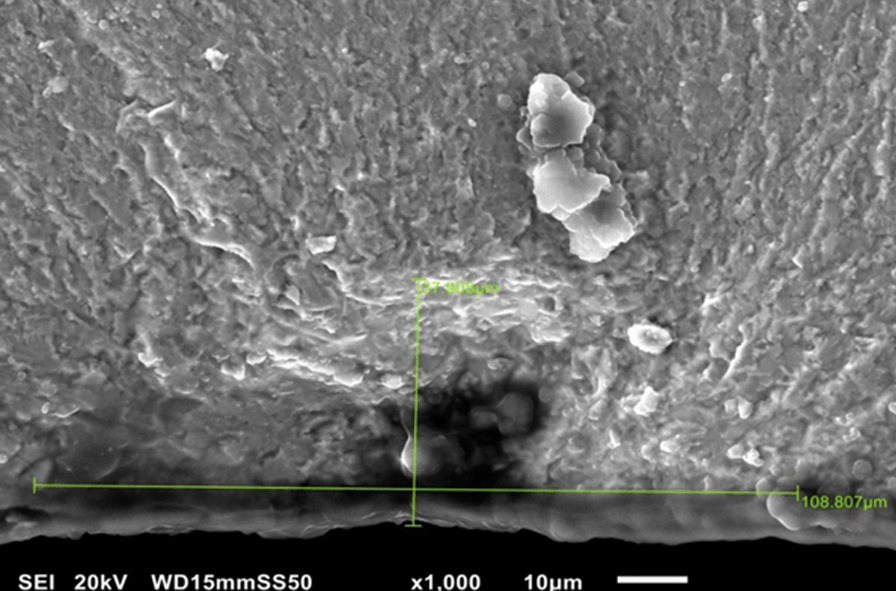
Scanning electron microscope image (× 1000) showing the measurement taking of the height (**a**) and width (**b**) of the critical size defect (cr)

All fractured specimens were cleaned in an ultrasonic bath for 10 min, dried in an electric oven at 100 °C for 4 min, gold sputter coated (S150B sputter coater; Edwards, Crawly, UK), and examined under a scanning electron microscope (XL30, Philips, Eindhoven, The Netherlands). The origin and direction of the fracture were identified and located followed by measuring the size of the critical defect (cr) (Fig. 2) that caused the fracture using the following formula [27]:

4 $${\text{cr }} = \sqrt {a.b}$$

where (cr) is the critical defect size in µm ($a$) is the height of the defect origin and ($b$) is its half-width in µm.

The fracture toughness (K_IC_) of fractured zirconia bars could be then calculated according to c and Fs values obtained using the following equation[27]:

5 $${\text{K}}_{{{\text{IC}}}} = {\text{Y}}.Fs\sqrt {cr}$$

where (K_IC_) is the fracture toughness in MPa.m^1/2^, Y is the geometry constant = 1.24 (assuming that there is a lack of pre-stresses at crack origin), and cr is the critical defect size in µm.

### Cyclic fatigue test

The other half of zirconia specimens received 10^6^ cycles, equivalent to 4 years of service in the patient’s mouth [28]. The piston on three balls set-up was immersed in a container of distilled water at 37 °C where each zirconia disc was subjected to fatigue testing using an ACTA type five-unit pneumatic fatigue tester (ACTA, Amsterdam, The Netherlands). The cycling loading was set at 40% of the mean initial biaxial flexural strength, previously obtained from the treated zirconia specimens for each material (n = 20) [26]. Cyclic loading was set to 40% to ensure the survival of zirconia discs. After completing the required cycles, zirconia discs were loaded to failure as previously described to evaluate the residual biaxial flexural strength.

### Weibull modulus

Weibull modulus (m) was calculated to determine the reliability of the materials according to Quinn and Quinn method [29]. This equation: ${\text{P}}(\upsigma ) = 1 - \exp ( - \sigma /\sigma_{0} )^{m}$ was used to calculate the (m) value. Where P(σ) is the fracture probability, σ is the flexural strength at a given P(σ), ${\sigma }_{0}$ is the characteristic strength at which 62.3% of the specimens are expected to fracture, and m is the Weibull modulus which is obtained by calculating the slope of the plot between 1n (1n 1/1 − P) versus σ [29]. Two-parameter Weibull distribution fit for the obtained biaxial flexural strength values was done by computer software (Weibull^++^ version 21, Reliasoft, Tucson, USA) using the maximum likelihood estimation method with a 95% confidence interval (CI) [30].

### Statistical analysis

Computer software (SPSS for Windows version 22.0 (SPSS Inc., Chicago, IL, USA)) was used to run all the statistical analyses of the obtained data. The normal distribution and homogeneity of variance were checked and verified for surface roughness parameters, fracture toughness, and biaxial flexural strengths data using Kolmogorov–Smirnov and Levene’s tests, respectively. The data were found to be normally distributed. Hence, one-way analysis of variance (ANOVA) was used to compare the mean surface roughness parameters and fracture toughness of the studied groups. However, the effect of different zirconia types, surface treatments applied, and fatigue was analyzed by three-way ANOVA and Tukey HSD post hoc test for multiple comparisons at a level of significance (α = 0.05).

## Results

**Fig. 3:**
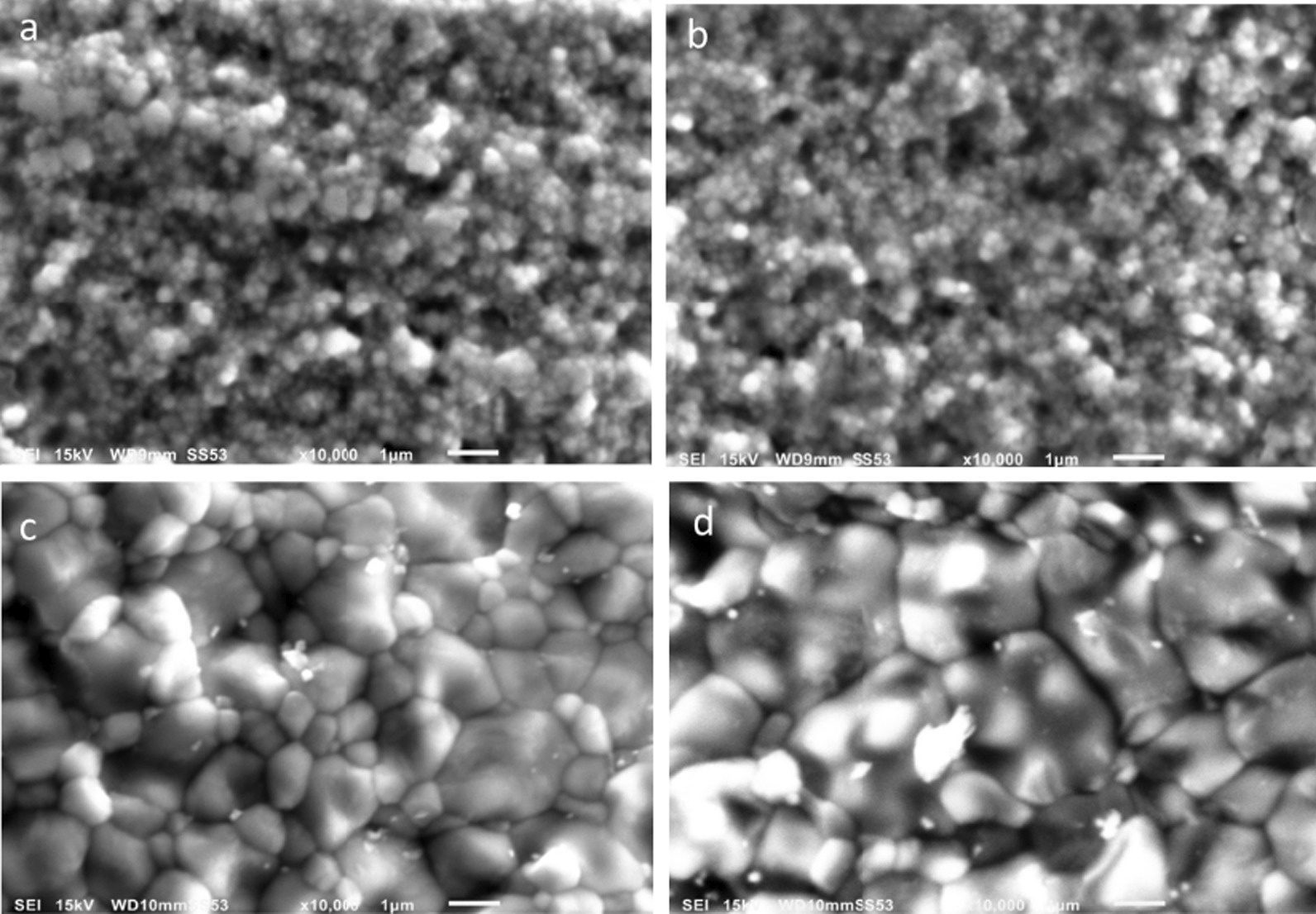
Scanning electron microscope images (× 10,000) showing homogenous round small grains of **a** Cercon and **b** Lava zirconia. However, larger cubic-shaped grains are noticed in HT, **c** BruxZir and **d** Katana zirconia

Examination of the internal structure of the tested zirconia revealed that low translucency zirconia was composed of submicroscopic round grains with an average size of 0.2–0.3 µm demonstrating homogenous and thick grain boundary regions (Fig. 3a and b). High-translucent zirconia showed a much larger grain size in a range of 1.2–1.6 µm with more refined grain boundary regions. Larger cubic grains ranging between 1.9 and 3.6 µm^2^ were frequently interrupted by smaller round grains (0.9–1.6 µm^2^) (Fig. 3c and d). However, Katana was characterised by more uniform larger grains compared to BruxZir zirconia.

Descriptive statistics and one-way ANOVA results of surface roughness parameters and fracture toughness are summarised in Table 2. Generally, there was no statistically significant difference (*P* > 0.05) in the surface roughness parameters (Ra. Rv, and Rp) between the as-sintered and APA groups regardless of the zirconia type used. However, surface grinding statistically (*p* < 0.001) increased the surface roughness parameters in all zirconia groups. There was no statistically significant difference (*p* > 0.05) in the fracture toughness between the Cercon and Lava (LT) or between BruxZir and Katana (HT) zirconia types in the as-sintered groups or the APA groups. Further, no significant difference was detected in ground groups regardless of the zirconia type. Low-pressure APA significantly increased the fracture toughness compared to the as-sintered groups in all zirconia types except for BruxZir; no significant difference was found (*p* > 0.05). Surface grinding significantly decreased (*p* < 0.001) the fracture toughness in all zirconia types with no statistical difference between them.

**Table 2:** Descriptive statistics of surface roughness parameters (µm), fracture toughness (K_IC_), and biaxial flexural strengths (MPa) before and after fatigue for different study groups

Materials	Surface treatment	Mean surface roughness ± SD (µm)			K_Ic_	Mean biaxial flexural strength ± SD (Mpa)	
		R_a_	R_v_	R_p_	MPa.m^1/2^	Initial	Residual
Cercon Base (LT)	As-sintered	1.56 ± 0.2 ^A^	8.1 ± 0.3 ^A^	6.3 ± 1.2 ^A^	5.6 ± 0.3 ^A^	1247.5 ± 25.8 ^A1^	1044.9 ± 34.4 ^A2^
	APA	1.8 ± 0.1 ^A^	9.8 ± 1.3 ^A^	7.9 ± 0.9 ^AB^	6.1 ± 0.25 ^B^	1451.5 ± 34.9 ^B1^	1228 ± 18.5 ^B2^
	Grinding	5.4 ± 0.09 ^BC^	14.2 ± 2.4 ^B^	9.7 ± 1.8 ^C^	4.1 ± 0.87 ^C^	909.7 ± 22.9 ^C1^	690.7 ± 18.3 ^C2^
Lava Frame (LT)	As-sintered	1.4 ± 0.07 ^A^	8.4 ± 1.4 ^A^	6.3 ± 0.8 ^A^	5.7 ± 0.37 ^A^	1271 ± 14.1 ^A1^	1059 ± 30.4 ^A2^
	APA	1.8 ± 0.04 ^A^	9.3 ± 0.7 ^A^	8.7 ± 0.9 ^BC^	6.0 ± 0.29 ^B^	1442.4 ± 24.3 ^B1^	1259 ± 24.6 ^B2^
	Grinding	6.0 ± 0.08 ^B^	13.1 ± 2.1 ^BC^	9.1 ± 1.6 ^C^	4.3 ± 0.92 ^C^	973.4 ± 23.9 ^D1^	668.9 ± 32.1 ^C2^
Katana (HT)	As-sintered	1.5 ± 0.04 ^A^	8.3 ± 1.3 ^A^	6.4 ± 1.1 ^A^	6.1 ± 0.19 ^B^	812.8 ± 27.9 ^E1^	698.6 ± 19.7 ^C2^
	APA	2.1 ± 0.06 ^A^	10.4 ± 1.2 ^A^	7.0 ± 0.9 ^AB^	6.6 ± 0.41 ^D^	860.3 ± 18.2 ^F1^	769.2 ± 8.1 ^D2^
	Grinding	4.1 ± 1.03 ^CD^	11.5 ± 2.9 ^AC^	8.7 ± 1.3 ^BC^	4.7 ± 1.1 ^C^	692.3 ± 16.8 ^G1^	601.3 ± 20.2 ^E2^
BruxZir (HT)	As-sintered	1.46 ± 0.08 ^A^	8.6 ± 2.1 ^A^	6.9 ± 1.4 ^A^	6.3 ± 0.22 ^BD^	833.5 ± 13.2 ^EF1^	739.2 ± 5.4 ^D2^
	APA	2.3 ± 0.07 ^A^	10.7 ± 1.6 ^A^	6.6 ± 1.2 ^A^	6.7 ± 0.35 ^D^	858.3 ± 17.1 ^F1^	764.3 ± 18.3 ^D2^
	Grinding	3.0 ± 0.2 ^D^	11.2 ± 2.7 ^AC^	8.5 ± 1.9 ^BC^	4.4 ± 0.68 ^C^	686.5 ± 13.2 ^G1^	599.2 ± 10.5 ^E2^

No statistical significance (*P* > 0.05) is indicated by the same superscript capital letter in columns when comparing different surface treatments for different types of zirconia and by the same superscript numbers when comparing initial vs residual mean biaxial flexural strength

The 3-way ANOVA results showed that all the independent variables (zirconia type, surface treatments, and cyclic fatigue) or their interactions significantly affected the biaxial flexural strength among the studied groups. The greatest influence was for the type of zirconia used (partial eta squared η_P_^2^ = 0.987) followed by surface treatments applied (η_P_^2^ = 0.980), and cyclic fatigue (η_P_^2^ = 0.940), while the interaction effect between surface treatments and cyclic fatigue had the lowest impact (η_P_^2^ = 0.046).

Low-pressure APA significantly increased (*p* < 0.001) the biaxial flexural strength compared to the as-sintered groups in all zirconia types except for BruxZir zirconia, while surface grinding significantly decreased (*p* < 0.001) the initial biaxial flexural strength in all studied zirconia types. Cyclic fatigue significantly decreased (*p* < 0.001) the initial biaxial flexural strength in all zirconia types regardless of the surface treatment employed.

In the as-sintered group, LT zirconia types (Cercon and Lava) showed closely similar initial (1247.5 ± 25.8 Mpa and 1271 ± 14.1 MPa, respectively) biaxial flexural strength results with no significant difference between them (*p* > 0.05). However, HT zirconia types (Katana and BruxZir) showed significantly lower initial biaxial flexural strength (812.8 ± 27.9 and 833.5 ± 13.2, respectively) with no significant difference between them (*p* > 0.05). In the APA group, no significant difference was found in the initial biaxial flexural strength between (Cercon and Lava) zirconia (1451.5 ± 34.9 and 1442.4 ± 24.3, respectively), and similarly between (Katana and BruxZir) zirconia (860.3 ± 18.2 and 858.3 ± 17.1, respectively). In the surface grinding group, no significant difference was found in the initial biaxial flexural strength between Katana and BruxZir (692.3 ± 16.8 Mpa and 686.5 ± 13.2 Mpa, respectively). In comparison, Lava zirconia showed a significantly higher (*p* < 0.05) mean initial biaxial flexural strength (973.4 ± 23.9 MPa) compared to Cercon (909.7 ± 22.9 MPa). A similar trend of comparing the effect of different surface treatments on the residual biaxial flexural strength of different zirconia types was observed, except for comparing the residual biaxial flexural strength between Katana and BruxZir zirconia (739.2 ± 5.4 and 680 ± 19.7, respectively); a significant difference was found (*p* < 0.001).

Low-translucent zirconia was more affected by cyclic fatigue compared to HT zirconia. Cyclic fatigue had decreased the initial biaxial flexural strength of Cercon and Lava zirconia by 17% in the as-sintered specimens, and by 16% and 13%, respectively in the APA group, while ground zirconia specimens showed a reduction of 25% and 32%, respectively in their initial biaxial flexural strength after cyclic fatigue. HT zirconia was more resistant to cyclic fatigue compared to LT zirconia. In the as-sintered group, the initial biaxial flexural strength of Katana and BruxZir was reduced by 14% and 12%, respectively, and by 11% in the APA group, while ground zirconia specimens showed a reduction of their initial biaxial flexural strength by 14% and 13%, respectively.

**Fig. 4:**
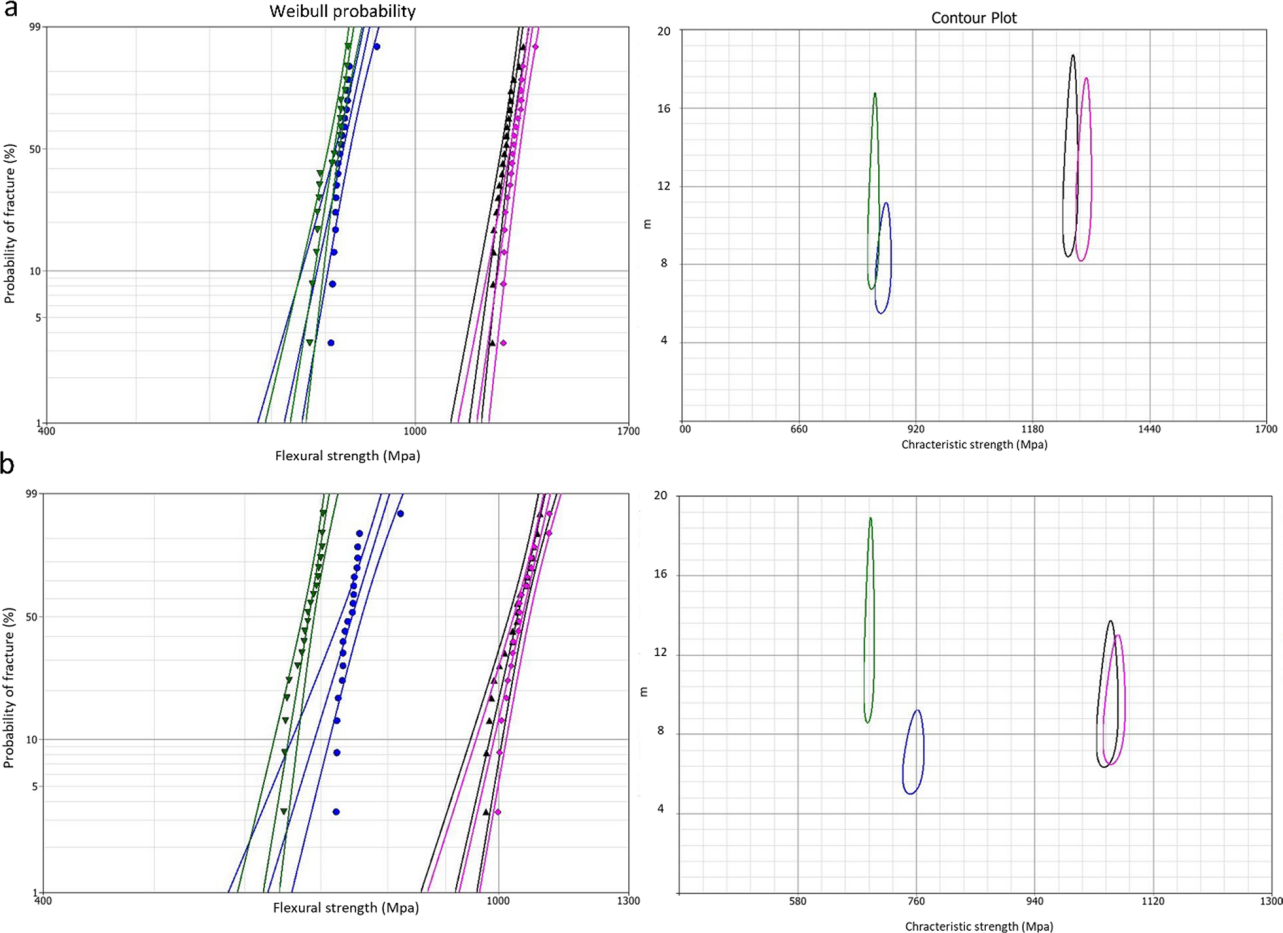
Graphs summarising Weibull probability and contour plots for different types of zirconia in the as-sintered group. For Weibull probability plots, the initial (**a**) and residual (**b**) biaxial flexural strength are presented on the x-axis and the probability of fracture on the y-axis, while for the contour plots, Weibull modulus (m) and characteristic strength (${\sigma }_{0}$) are presented on y and x axes, respectively. Two-parameter Weibull distribution, with 95% CI, was applied for Lava (pink color), Cercon (black color), BruxZir (Blue color), and Katana (green color). The central line for each group represents the probability line, while the top and bottom lines represent 95% CI bounds

**Fig. 5:**
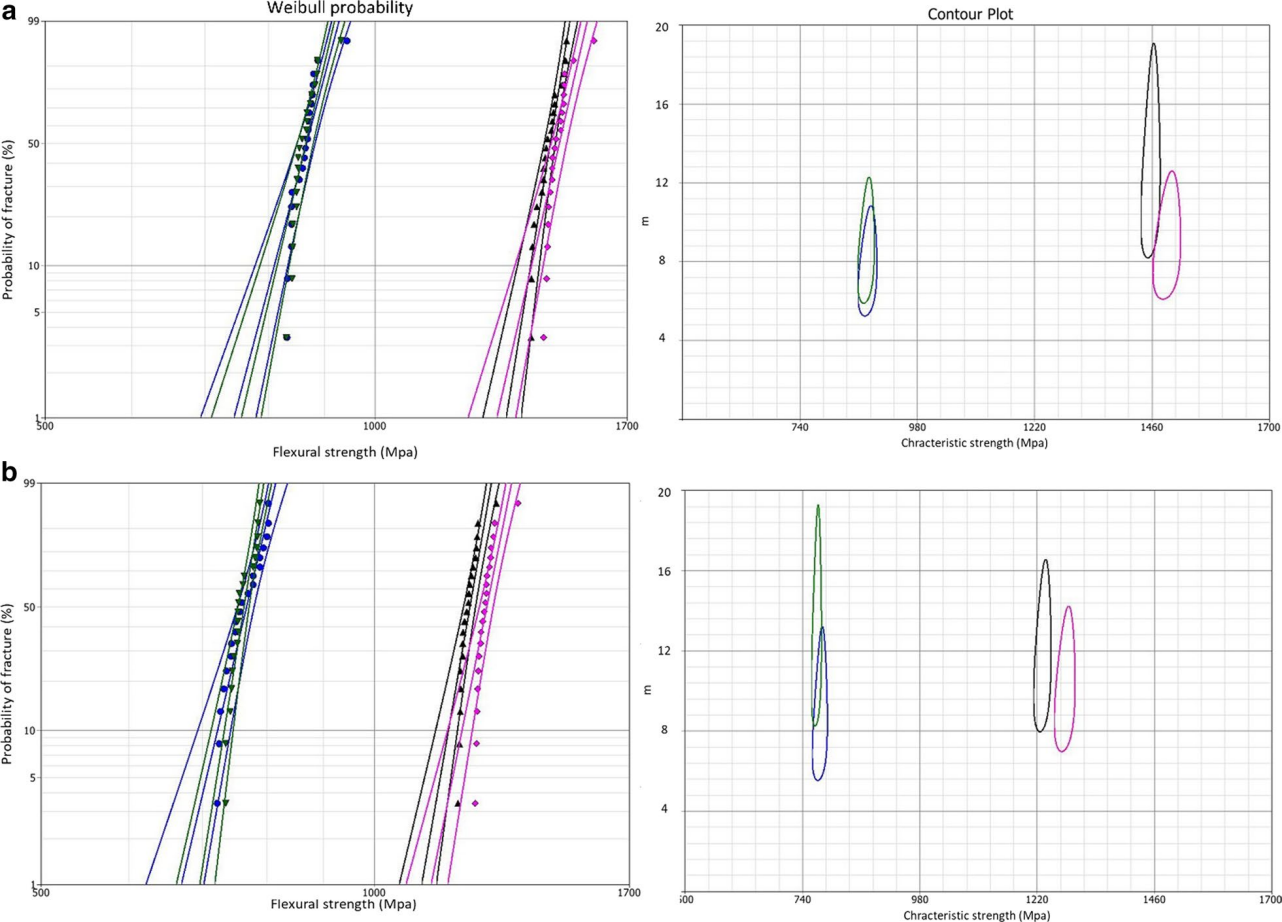
Two- parameter Weibull analysis of the initial (**a**) and residual (**b**) biaxial flexural strength for the blasted zirconia groups

**Fig. 6:**
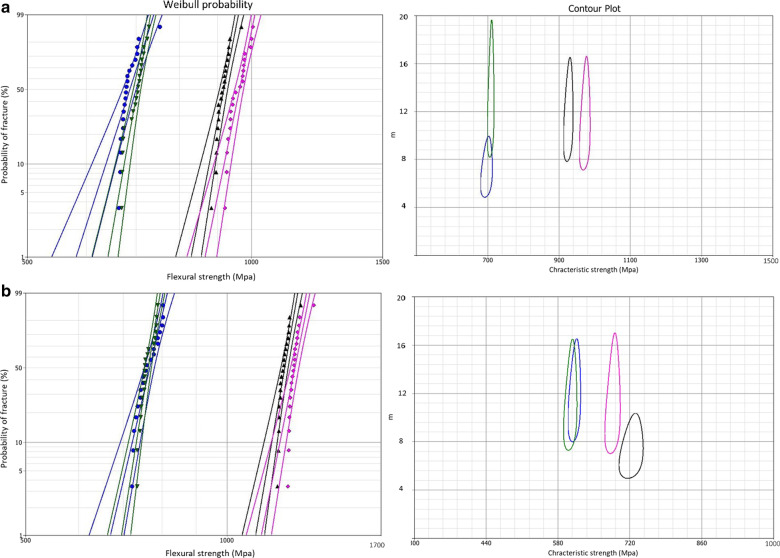
Two-parameter Weibull analysis of initial (**a**) and residual (**b**) biaxial flexural strength for different zirconia types underwent grinding surface treatment

Weibull parameters of initial and residual biaxial flexural strength values for all studied groups are presented in Figs. 4, 5, and 6 and are summarised in Table 3. The obtained Weibull moduli (m) and characteristic strength (${\sigma }_{0})$ for initial and residual biaxial flexural strength of all groups are plotted in contour plots. The non-overlapping between the bounds in contour plots indicates significant differences in m and ${\sigma }_{0}$ between the compared groups. Generally, Weibull moduli and charactersitic strength values were lower for the residual, compared to the initial, mean biaxial flexural strength data indicating less reliable materials after cyclic fatigue.

**Table 3:** Two-parameter Weibull modulus (m) for different zirconia groups and their characteristic strength (${\sigma }_{0})$ in Mpa

Materials	Surface treatment	Weibull modulus (m)		Characteristic strength $(\sigma_{{\text{o}}} )$	
		Initial	Residual	Initial	Residual
Cercon Base (LT)	As-sintered	14.8	10.1	1263.6	1050.3
	APA	12.4	11.6	1457.2	1230.7
	Grinding	13.8	6.9	925.9	722.4
Lava Frame (LT)	As-sintered	13.1	9.2	1292.7	1060.5
	APA	9.3	8.2	1490.5	1276.4
	Grinding	11.6	9.8	972.4	686.5
Katana (HT)	As-sintered	10.7	9.3	825.2	688.3
	APA	8.6	7.3	875.1	768.8
	Grinding	13.9	11.2	708.3	604.2
BruxZir (HT)	As-sintered	8.1	7.2	846.9	755.7
	APA	8.3	7.1	877.9	775.8
	Grinding	7.1	6.7	696.4	611.9

## Discussion

This study aimed to evaluate the biaxial flexural strength, fracture toughness, and fatigue resistance of two HT and LT zirconia after various surface treatments. A significant difference was found between the studied groups, and therefore the null hypothesis was rejected.

Zirconia is a category of brittle ceramics which are more sensitive to tensile than compressive forces [31]. Mechanical properties such as flexural strength and fracture toughness can characterise the mechanical performance of zirconia. Among the well-known flexural strength testing approaches, piston-on-3-ball biaxial flexural strength test was applied as recommended by ISO 6782. Three-point and 4-point flexural strength testing are other potential approaches to characterise the mechanical properties of zirconia. However, these approaches employ the use of bar-shaped specimens that are affected by edge defects in the bar design, while biaxial flexural strength testing requires disc-shaped specimens supported on three metal spheres and a load applied to the centre of the specimen [32].

Surface grinding and APA were applied in the current study as mechanical surface treatments to zirconia as they are routinely performed in the clinical situation to improve the resin bonding to tooth structure or to the veneering porcelain [33]. Further, grinding is commonly done during fit corrections of zirconia frameworks [12, 21, 34]. However, APA was employed, in a low-pressure mode, to decrease the possible critical surface flaws that act as stress concentration sites and potential crack origins under loads [35–38].

Low APA and surface grinding increased the surface roughness parameters with a statistical significance detected in the latter. These findings are in agreement with previous work [25, 39, 40]. Hight translucent zirconia showed higher surface roughness parameters in the APA group compared to LT zirconia. During APA, zirconia grains are pulled out [22]. The larger grains of HT zirconia could have left a more considerable defect behind, and hence a higher surface roughness was found compared to the smaller grain size of LT zirconia, in agreement with a previous study [40]. Grinding produced a more irregular surface with deep grooves that acted as stress concentration areas and in turn increased all roughness parameters (R_a_, R_v,_ and R_p_) and decreased both initial and residual flexural strengths. Özcan et al. [41] concluded that APA using 50 μm created more surface roughness, and reduced the biaxial flexural strength and Weibull modulus of the examined tetragonal LT zirconia. On the contrary, the present study has shown that low-pressure APA increased both biaxial flexural strength and Weibull modulus due to the transformation toughening property of tetragonal zirconia, which is consistent with several other studies [42–44]. It has to be mentioned that the pressure applied in this study was much weaker, as recommended by several manufacturers and reports [44–46].

Low translucency zirconia had a significantly higher initial biaxial flexural strength compared to HT zirconia. Several studies have related the increase in grain size to the decrease in flexural strength [47, 48]. Accordingly, it was expected for HT zirconia to have less flexural strength than LT zirconia [49]. The presence of smaller grains helped to maintain a relatively high flexure strength compared to other all-ceramic materials [50]. The grain size of 0.9 μm to 1.4 μm can increase the fracture strength linearly from 650 MPa to 1000 Mpa [51]. The combination of various grain sizes allowed HT zirconia to be applied in stress-bearing areas [47, 52]. Reducing the size of the grain beyond 0.5 µm is known to increase the mechanical properties, which came at the expense of translucency [53].

High translucency zirconia revealed a higher fatigue resistance, compared to LT zirconia, as it was associated with a lower percentage of reduction in residual strength due to its internal structure, larger grain size, and refined grain boundaries. Some studies found that the percentage of transformation toughening that hinders crack propagation in LT tetragonal zirconia was much higher than HT cubic zirconia [43–48], so it was expected for LT zirconia to be more resistant to fatigue compared to HT zirconia. Such a finding cannot be attributed to phase transformation alone. Still, it is directly related to the internal structure of the materials and the mechanism that larger grains might interrupt the propagation of crack tips [54–58].

The current study showed that the fracture toughness of HT zirconia was higher than that of LT zirconia which can be attributed to the larger grain size of the first [59] as there is a strong direct relationship between fracture toughness and grain size. High translucency zirconia was associated with relatively smaller critical crack sizes compared to LT zirconia. Rougher crack surfaces indicated that cracks traveled at grain boundary regions instead of splitting the grains, especially in its first stages. Larger grains mean longer crack paths, which could explain the higher fracture toughness observed for HT zirconia. Another study stated that the fracture toughness of zirconia is closely related to the transformation toughening ability as the transformation process itself helped in dissipating the energy associated with crack propagation [4]. Nevertheless, an optimised internal structure is of prime importance as transformation toughening is a process limited to the presence of stresses, and regions, outside the stress field, will not benefit from this process.

Weibull distribution is commonly used in life prediction analysis of brittle materials such as zirconia [60]. For long-term clinical success, a material with a higher Weibull modulus is more advantageous than a stronger material with a lower value. The Weibull modulus of dental ceramics was found to range from 5 to 15 [61]. In the current study, Weibull modulus values ranged from 6.7 to 14.8, indicating different degrees of fracture probability under clinical conditions.

The presence of deep surface defects introduced by grinding had a direct effect on the reduction of residual strength associated with lower (m) values as reported by previous works [61, 62]. Further studies are needed to elaborate on crystallographic changes associated with the two materials. Low-pressure airborne-particle-abraded specimens showed the lowest (m) values. In contrast, ground specimens showed higher values than the as-sintered zirconia, which is consistent with the results of other studies that reported no difference between Weibull modulus values of different surface treatments indicating that the flaw size distribution was very similar [63–65].

The limitations of the current study can be the use of cylindrical specimens design that do not simulate the anatomical crowns in clinical situations. However, the cylindrical design was easier to standardise for all tested specimens, so that the results can be fairly compared between the tested groups and a conclusion can be precisely drawn. Other limitations can be the use of two LT and HT zirconia types in the study. Supra-high translucent zirconia and ultra-high translucent zirconia are other common brands of PSZ zirconia that should be tested to increase the external validity of the results. Further, crystallographic analysis of treated zirconia types was not done. Assessment of crystalline changes to zirconia after various surface treatments will give a more in-depth understating of how much they can trigger t-m transformation.

## Conclusion

Low translucency zirconia has higher biaxial flexural strength, yet with lower fracture toughness and fatigue resistance, compared to HT zirconia. Low-pressure APA has significantly increased the biaxial flexural strength in all zirconia groups except BruxZir. Grinding was deteriorating to biaxial flexural strength and fracture toughness in all zirconia types. Cyclic fatigue has significantly decreased the biaxial flexural strength and reliability of HT and LT zirconia.

## Data Availability

The datasets used and/or analysed during the current study are available from the corresponding author on reasonable request.

## Abbreviations

Y-TZP: Yttria tetragonal zirconia polycrystal
APA: Air-borne particle abrasion
PSZ: Partially stabilized zirconia
HT: High translucency
LT: Low transclucency
ANOVA: Analysis of variance
